# Shared Microbial Taxa Respond Predictably to Cyclic Time-Varying Oxygen Limitation in Two Disparate Soils

**DOI:** 10.3389/fmicb.2022.866828

**Published:** 2022-06-02

**Authors:** Steven J. Hall, Wenjuan Huang, Stephanie A. Napieralski, Eric Roden

**Affiliations:** ^1^Department of Ecology, Evolution and Organismal Biology, Iowa State University, Ames, IA, United States; ^2^Department of Geoscience, University of Wisconsin-Madison, Madison, WI, United States

**Keywords:** anaerobic, anoxic, methane, oxygen, 16S rRNA, redox, wetland, iron reduction and oxidation

## Abstract

Periodic oxygen (O_2_) limitation in humid terrestrial soils likely influences microbial composition, but whether communities share similar responses in disparate environments remains unclear. To test if specific microbial taxa share consistent responses to anoxia in radically different soils, we incubated a rainforest Oxisol and cropland Mollisol under cyclic, time-varying anoxic/oxic cycles in the laboratory. Both soils are known to experience anoxic periods of days to weeks under field conditions; our incubation treatments consisted of anoxic periods of 0, 2, 4, 8, or 12 d followed by 4 d of oxic conditions, repeated for a total of 384 d. Taxa measured by 16S rRNA gene sequences after 48 d and 384 d of experimental treatments varied strongly with increasing anoxic period duration, and responses to anoxia often differed between soils at multiple taxonomic levels. Only 19% of the 30,356 operational taxonomic units (OTUs) occurred in both soils, and most OTUs did not respond consistently to O_2_ treatments. However, the OTUs present in both soils were disproportionally abundant, comprising 50% of sequences, and they often had a similar response to anoxic period duration in both soils (*p* < 0.0001). Overall, 67 OTUs, 36 families, 15 orders, 10 classes, and two phyla had significant and directionally consistent (positive or negative) responses to anoxic period duration in both soils. Prominent OTUs and taxonomic groups increasing with anoxic period duration in both soils included actinomycetes (*Micromonosporaceae*), numerous *Ruminococcaceae*, possible metal reducers (*Anaeromyxobacter*) or oxidizers (*Candidatus* Koribacter), methanogens (*Methanomicrobia*), and methanotrophs (*Methylocystaceae*). OTUs decreasing with anoxic duration in both soils included nitrifiers (*Nitrospira*) and ubiquitous unidentified *Bradyrhizobiaceae* and *Micromonosporaceae*. Even within the same genus, different OTUs occasionally showed strong positive or negative responses to anoxic duration (e.g., *Dactylosporangium* in the *Actinobacteria*), highlighting a potential for adaptation or niche partitioning in variable-O_2_ environments. Overall, brief anoxic periods impacted the abundance of certain microbial taxa in predictable ways, suggesting that microbial community data may partially reflect and integrate spatiotemporal differences in O_2_ availability within and among soils.

## Introduction

Surface soils of humid biomes experience variation in oxygen (O_2_) availability over multiple spatial and temporal scales. The existence of mm-scale anoxic microsites within well-drained bulk oxic soils has been known for decades (Sexstone et al., 1985; von Fischer and Hedin, 2002). The anoxic soil volume varies over time as a function of physical O_2_ supply, labile carbon (C) availability, and biological O_2_ demand (Keiluweit et al., 2016). For instance, O_2_ in the pores of surface soils may vary from atmospheric levels to below detection over timescales of days to weeks to months (Silver et al., 1999; Liptzin et al., 2011; Hall et al., 2013; Huang and Hall, 2017; O’Connell et al., 2018). Availability of O_2_ has been proposed as one of the most important factors structuring soil microbial community composition (Fierer, 2017), as implied by differences in oxidoreductase genes among habitats (Ramírez-Flandes et al., 2019). Indeed, field measurements demonstrated that proxies for O_2_ may predict spatial or depth variation in microbial community composition within individual sites (Peralta et al., 2014; Lipson et al., 2015). An increasing number of studies has explicitly investigated the impact of temporal O_2_ variation over timescales of hours to weeks on microbial community composition, but responses of particular microbial groups or taxa are often difficult to generalize among studies (Pett-Ridge and Firestone, 2005; DeAngelis et al., 2010; Frindte et al., 2016; Mejia et al., 2016; Randle-Boggis et al., 2018; Winkler et al., 2019; Pronk et al., 2020). These differences raise the question of whether microbial communities or individual microbial taxa from disparate soils might share consistent responses to time-varying anoxic conditions, or whether differences in other soil characteristics, such as pH, organic matter availability, mineralogy, or other site-specific factors might override the effects of anoxia. Diverse terrestrial soils appear to share a core group of common and widespread bacterial taxa, which vary in abundance among habitats as a function of pH, aridity, and plant productivity (Delgado-Baquerizo et al., 2018). Similarly, temporal variation in O_2_ availability might also drive consistent responses in the relative abundance of core bacterial groups across diverse habitats.

Cyclic anoxic events may influence microbial community composition by maintaining generalist taxa tolerant of both aerobic and anaerobic conditions, and the relative abundance of these taxa might reflect the redox history of a given soil. In fact, previous experiments suggested that the microbial community composition of soils that routinely experience high-frequency (hours–days) O_2_ variability may respond little to short-term anoxic events because of adaptation to O_2_ fluctuations. In tropical rainforest soils, experimental 4-d anoxic/oxic fluctuations maintained bacterial composition most similar to *in-situ* communities, whereas communities from static oxic or anoxic treatments diverged (Pett-Ridge and Firestone, 2005; DeAngelis et al., 2010). More taxa were active under fluctuating than static redox treatments, indicating that redox variability could maintain diversity (DeAngelis et al., 2010). However, community change might be expected to occur if the duration of anoxic or oxic events exceeds the capacity of sensitive organisms to persist through unfavorable conditions (Pett-Ridge and Firestone, 2005). Bacterial communities incubated under 5-week anoxic/1-week oxic cycles for 1 y diverged from initial conditions in one rice paddy soil but not another (Winkler et al., 2019). Bacteria exposed to 4-week drained and flooded cycles in a synthetic soil did not differ from a static control (Pronk et al., 2020), but repeated 2-week flooding events altered pasture soil communities (Randle-Boggis et al., 2018). Overall, responses of soil microbial communities to experimental manipulations of O_2_ availability have been mixed, and it is not clear whether community responses to cyclic anoxic events of increasing duration are generally consistent within or among soils.

More broadly, we lack an understanding of whether consistent groups of microbial taxa share responses to short-term anoxic periods across disparate environments. Anoxic sewage digestors from disparate locations shared a core suite of microbes (Rivière et al., 2009), but to our knowledge, a suite of common and abundant bacteria correlated with periodic O_2_ deficiency in soil has not been identified. While there is increasing evidence that even closely related microbes may respond differently to environmental factors (e.g., Bier et al., 2015; García-García et al., 2019; Yu et al., 2021), some habitat preferences and functional attributes may remain conserved at relatively high taxonomic rank (Fierer et al., 2007; Philippot et al., 2010). Indeed, we might expect this reasoning to apply for O_2_ availability, given its central role as an electron acceptor for some organisms and toxin for others. For example, nitrifiers in phylum *Nitrospirae* require O_2_ for nitrite oxidation (Koch et al., 2015), whereas methanogens in phylum *Euryarchaeota* are thought to be strict anaerobes (Whitman et al., 2014). Nevertheless, taxa within these groups might differ in their sensitivity to O_2_. Nitrifiers may persist after months of anoxia (Pett-Ridge et al., 2006), possibly by exploiting alternative metabolisms (Koch et al., 2015), and methanogens may persist even in desert soils (Peters and Conrad, 1995). Members of some higher-level taxonomic groups clearly differ in their O_2_ requirements: for example, *Actinobacteria* comprise many aerobic organisms that decompose lignocellulose, but others are facultative or perhaps obligate anaerobes (Wellington and Toth, 1994; Barka et al., 2016). These findings highlight uncertainty about which microbial groups might respond most strongly to variation in soil O_2_ availability, and over what timescales.

**Figure 1:**
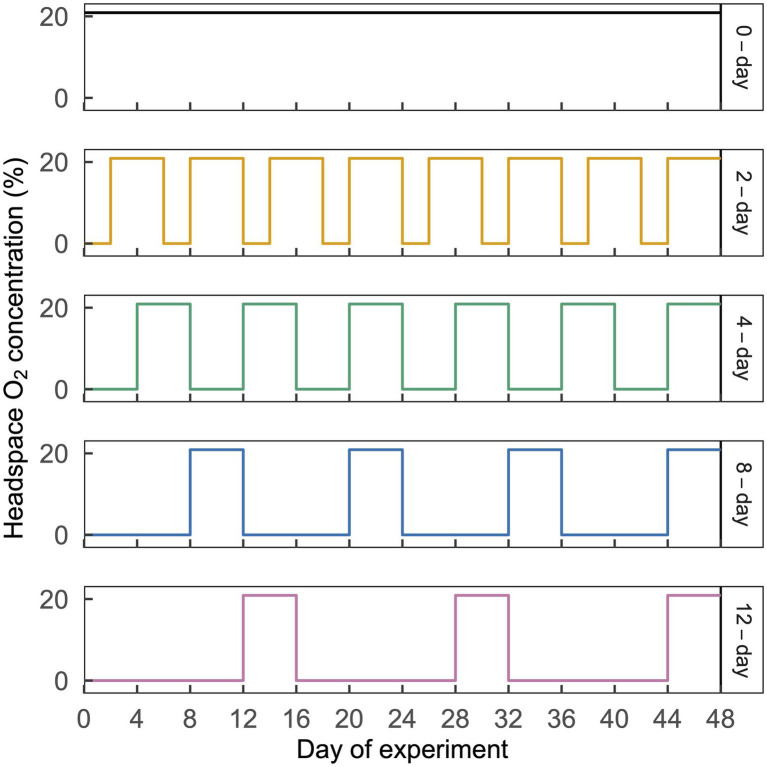
Illustration of the cyclic time-varying O_2_ treatments imposed in this study. Headspace O_2_ concentration is shown on the left y-axis and treatment names are shown on the right y-axis, with line colors corresponding to the ordinations in Figure 3. Each treatment is shown in a separate horizontal panel. The 2-day, 4-day, 8-day, and 12-day treatments received an N_2_ headspace for a period of 2, 4, 8, or 12 d, which was followed by 4 d of CO_2_-free air. A representative 48-d time period is shown above; this same pattern was repeated a total of eight times over 384 d. This figure is modified from Huang et al. (2021).

As a proof of concept to test how microbes may respond to cyclic anoxic events of differing duration (days–weeks) in greatly contrasting habitats, we conducted laboratory experiments with two terrestrial soils: a tropical rainforest Oxisol and a temperate cropland Mollisol. These soils were chosen because they both experience anoxic events of days to weeks under field conditions as a consequence of high clay content, carbon availability, and episodically high moisture. They also differ greatly in key properties (Table 1) such as pH, mineralogy, climate, and vegetation (Huang et al., 2020b, 2021). To quantify relationships between O_2_ availability and community composition, we exposed soils to repeated anoxic/oxic cycles with anoxic periods of 0, 2, 4, 8, or 12 d, each of which was followed by a consistent 4-d oxic period; these cycles were repeated for a total of 384 d (Figure 1). The anoxic period durations were chosen to bracket a range of anoxic events that may occur in these soils following precipitation or ponding events, or periods of C influx from litterfall or tillage, as evidenced by measurements of moisture, O_2_, Eh, and/or redox-sensitive iron species (Silver et al., 1999; Hall et al., 2013; Huang and Hall, 2017; Barcellos et al., 2018; O’Connell et al., 2018; Martin et al., 2019; Yu et al., 2021). The experiment was conducted over 384 d to allow greater capacity to detect effects which might otherwise be obscured by legacies of recent environmental conditions at the time of soil sampling.

**Table 1:** Key attributes of soils used in this experiment.

Soil	Ecosystem type	pH	Soil organic carbon (mg C g^−1^)	C:N ratio	Mean annual temperature (C)	Mean annual precipitation (mm)	Clay (%)	Silt (%)	Sand (%)	Pedogenic iron (mg g^−1^)
Oxisol	Humid tropical forest	5	44.8	10.8	24	3,800	39	53	8	52
Mollisol	Temperate cropland	8.3	35.1	11.3	9	820	38	40	22	5

Biogeochemical data from this experiment were published previously in a paper where we tested impacts of cyclic anoxic conditions on organic matter decomposition and the representation of these processes in a mechanistic ecosystem C-cycling model (Huang et al., 2021). Here, we report microbial community data from the same experiment to understand how periodic anoxic conditions impacted microbial community composition in these soils. Biological replicates were destructively sampled for DNA extraction and sequencing of the 16S rRNA region after 0, 48, and 384 d. We analyzed DNA rather than RNA because we sought to characterize the time-integrated responses of communities to the experimental treatments (including dormant, active, and recently deceased organisms) as opposed to short-term changes in activity, which we would expect to vary markedly within an individual treatment over a single oxic/anoxic cycle (DeAngelis et al., 2010; Blazewicz et al., 2013). We hypothesized (1) that community composition would change predictably as a function of increasing anoxic period duration, and (2) that a core group of taxa would respond similarly to anoxic period duration in both soils.

## Materials and Methods

### Experimental Design

The rainforest Oxisol was sampled from a per-humid lower montane site in the Luquillo Experimental Forest, Puerto Rico, and the cropland Mollisol was sampled from a central Iowa field under long-term corn/soybean (*Zea mays*/*Glycine max*) cultivation. General properties of these soils are listed in Table 1 and were further described in a companion study (Huang et al., 2021). Replicates from each soil were subjected to each of five headspace treatments, consisting of anoxic periods of 0, 2, 4, 8, or 12 d, each followed by a 4-d oxic period; these cycles were repeated for the duration of the experiment (384 d). The total number of anoxic/oxic cycles varied among treatments according to the length of the anoxic phase. There was a total of 64, 48, 32, or 24 cycles for the 2-day, 4-day, 8-day, or 12-day treatments.

Each experimental replicate comprised 5 g soil (dry mass equivalent, amended with water to field capacity) incubated inside a separate glass jar (0.95 l) with a gas-tight aluminum lid sealed with a Viton gasket and two butyl septa for headspace flushing and venting as described below. Field moisture capacity measured 1.01 g H_2_O and 0.46 g H_2_O per g dry soil for the Oxisol and Mollisol, respectively (Huang et al., 2020b). Because labile C may gradually become depleted during soil incubations (Chacon et al., 2006), we amended soils with ground litter at the beginning of the experiment to simulate a C-rich surface soil microsite. Each replicate of each soil was amended with 0.5 g of dried and ground aboveground litter from *Andropogon gerardii* (a perennial C_4_ grass) to ameliorate short-term C limitation and to simulate conditions that prevail in C-rich surface soil microsites. This plant was not present in either of these soils prior to the experiment.

Anoxic or oxic treatments were imposed by manually replacing the jar headspace with humidified dinitrogen gas (N_2_) or humidified CO_2_-free air, respectively, at 2-d intervals throughout the experiment. We chose to directly manipulate headspace O_2_ content, rather than controlling O_2_ availability indirectly *via* soil moisture, to achieve a more precise temporal gradient of O_2_ availability independent of changes in moisture (McNicol and Silver, 2014). There is a substantial literature on responses of soil microbial communities to dry/wet cycles (e.g., Placella et al., 2012) that is not directly relevant to this study, because anoxic conditions are not necessarily achieved following wet-up of dry soils, and because traits related to drought tolerance/resuscitation may not be directly relevant to O_2_ dynamics in soils from humid climates. Gas was flushed through each jar at >500 ml min^−1^ for 15 min *via* a distribution manifold connected to each jar by piercing a septum with a needle; jars were vented through a separate needle placed through the other septum. Needles were removed and jars were left sealed for 2 d, at which point the headspace gas was subsampled by syringe for CO_2_ and CH_4_ measurements (Huang et al., 2021), and jars were flushed again with the appropriate gas (N_2_ or CO_2_-free air). The logistical requirements of the headspace flushing regime limited the number of sacrificial replicates which could be included in the experiment. Water vapor lost during flushing was measured gravimetrically and periodically replenished at the end of an oxic period. The ratio of soil mass to headspace volume was chosen so that CO_2_ concentrations were always <6,000 ppm during oxic treatment phases. Analyses of CO_2_ in control jars (no soil) indicated that leaks were negligible. Mass balance indicates that any dissolved porewater O_2_ present during the oxic periods would be rapidly depleted by respiration during the anoxic periods. Porewater saturated with O_2_ at 23°C at 300 m (8.3 mg O_2_ L^−1^) contains 0.11–0.26 μmol O_2_ per g dry soil mass equivalent, given the moisture values listed above. Given observed mean CO_2_ + CH_4_ emissions of 3.3 μmol C g^−1^ d^−1^ (Huang et al., 2021), any remaining porewater O_2_ would be depleted within minutes to hours after the jar headspace was flushed with N_2_. Increased CH_4_ production following flushing with N_2_ (Huang et al., 2021) also indicates that anoxic conditions were achieved.

During the incubation, CO_2_ and CH_4_ production declined slowly over time in all treatments as available C was depleted (Huang et al., 2021). At the end of the experiment, CO_2_ + CH_4_ production was several-fold lower than at the beginning of the experiment, but an asymptote in cumulative decomposition was not observed, and analyses of the masses and isotope composition of cumulative CO_2_ and CH_4_ emissions showed that more than half of the added litter C and > 85% of the initial soil C remained (Huang et al., 2021). ^13^C NMR analyses indicated that much of this remaining C was contained in carbohydrates, and that C molecular composition was similar among treatments (Huang et al., 2020b, 2021). The sum of organic C decomposed to CO_2_ and CH_4_ was statistically equivalent among many of the headspace treatments and differed by <20% among all treatments from a given soil type (Huang et al., 2021). Therefore, we propose that differences in C availability *per se* were not likely to be the primary drivers of changes in community composition among headspace treatments.

### Data Analysis

We extracted genomic DNA from replicate subsamples after 0, 48, and 384 d (*n* = 3, 3, or 5, respectively per soil/headspace treatment; Supplementary Table S2) using MoBio Powersoil extraction kits (Maryland, United States). More replicates were available for analysis at 384 d than at the other timepoints because all of the replicates used for gas flux analysis (Huang et al., 2021) were sacrificed at that point; at 0 and 48 d, separate replicates not used for gas measurements were used for DNA analysis. Replicates were each incubated in separate jars. DNA was submitted to the University of Wisconsin-Madison Biotechnology Center for Illumina MiSeq 2×300 paired end sequencing of the V4 region of 16S rRNA; methodological details are described in the Supplementary Material. Raw sequences were deposited in the NCBI Sequence Read Archive under accession PRJNA693044. Sequences were processed with Quantitative Insights in Microbial Ecology (QIIME) 1.9.1 (Caporaso et al., 2010b). Prior to assembly of paired end sequences, low quality and ambiguous sequences were removed using the default QIIME parameters. Chimeric sequences were identified *via de novo* and reference based detection using usearch61 (Edgar, 2010). Operational taxonomic units (OTUs) were identified by *de novo* clustering (0.97 threshold) and were aligned against the SILVA database (Quast et al., 2013) using PyNAST (Caporaso et al., 2010a) to assign taxonomy.

Sequencing depth varied from 34,382 to 54,902 reads (median = 44,081) and two samples with <30,000 reads were discarded prior to subsequent analyses (Supplementary Table S2). Read counts did not vary systematically among headspace treatments, soils, or timepoints. We identified 40,345 OTUs in the initial dataset, which was used for calculation of diversity metrics prior to trimming rare taxa (McMurdie and Holmes, 2013). For community analyses, we discarded OTUs occurring in <3 samples and normalized counts by relative abundance. We conducted community analyses in R using phyloseq (McMurdie and Holmes, 2013) 1.28.0 and vegan 2.5.5 (Oksanen et al., 2019). We used distance-based redundancy analysis ordination using the “capscale” function with sampling date and anoxic duration as numeric predictors and tested their significance by permutation. To test responses of individual taxonomic groups to anoxic duration, we used differential expression analysis based on the negative binomial distribution implemented with DESeq2 1.24 (Love et al., 2014), correcting values of *p* for multiple comparisons using the Benjamini and Hochberg method. In separate DESeq analyses, we used OTUs, families, orders, classes, and phyla as response variables. Anoxic duration was coded as a continuous numeric predictor (regression design) such that the response of each OTU indicates the mean log2-fold change per day of anoxic duration. To test whether microbial groups had shared responses to anoxic duration in both soils, we coded direction of response (increase or decrease) of log2-fold change values for shared groups across different taxonomic levels, and used chi-squared tests to assess whether the overall direction of treatment response was significantly shared between soils at a given taxonomic level.

The replicates extracted for DNA at 384 d were also used for CO_2_ and CH_4_ measurements throughout the experiment (Huang et al., 2021), enabling us to assess relationships between cumulative CO_2_ and CH_4_ production and 16S composition at 384 d after accounting for headspace treatments and soil type. We used a permutational multivariate ANOVA on a Bray-Curtis distance matrix, with cumulative CO_2_, cumulative CH_4_, headspace treatment, and soil type as predictor variables. This analysis was conducted using the “adonis2” function in vegan 2.5.5 (Oksanen et al., 2019); we report the marginal effect of each model term.

## Results

### Community Responses to Anoxic Duration

**Figure 2:**
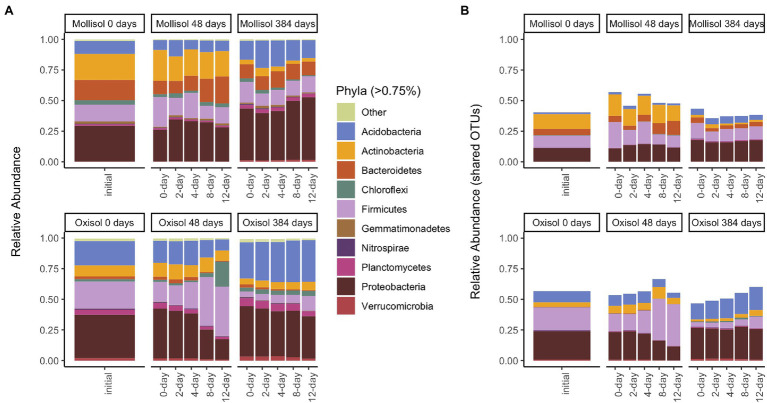
Mean sequence relative abundance shaded by phylum and plotted by experimental treatment and sampling date, including all OTUs **(A)**, or only OTUs that occurred in >2 samples in both soils **(B)**. Phyla comprising >0.75% of sequences in a given treatment are labeled (33 additional phyla are denoted “Other”). Panels represent sampling timepoints for each soil (0, 48, and 384 d), and bars within a panel represent means of headspace treatments (0, 2, 4, 8, or 12 d of anoxic conditions alternating with 4 d of oxic conditions).

**Figure 3:**
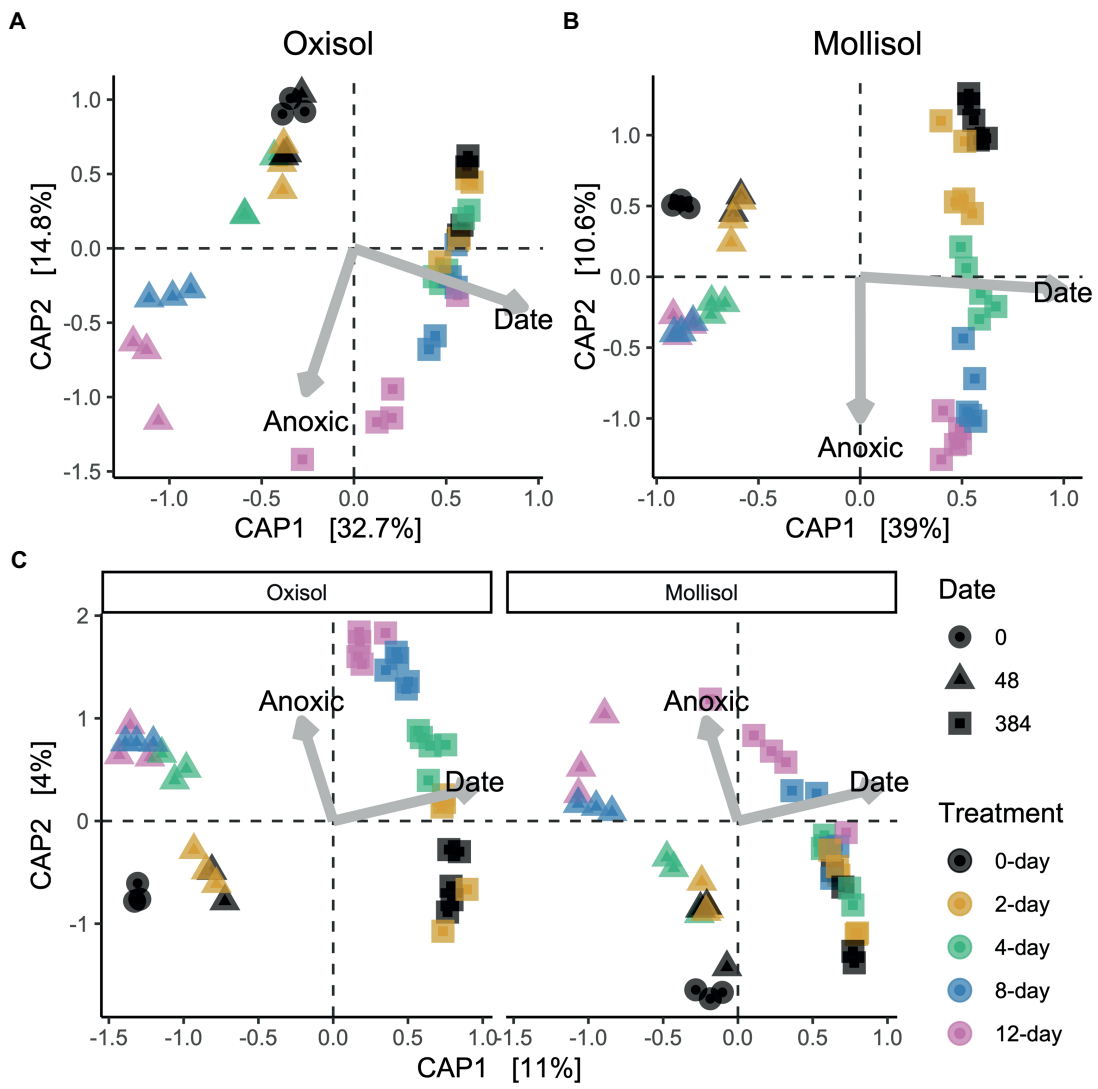
Distance-based redundancy analysis (dbRDA) ordinations of microbial communities in the Oxisol **(A)** or Mollisol **(B)**, and a combined ordination with data from both soils, where only OTUs present in both are included **(C)**, and soils are shown in separate panels for clarity (axis scores for the left and right panels of **(C)** are for the same ordination). CAP1 and CAP2 denote the first and second axes of the dbRDA ordination and values in brackets indicate variation explained by each axis. Scale values indicate correlation coefficients of numerical predictors (sampling date and anoxic treatment duration) with the respective axis.

As expected, the Oxisol and Mollisol differed substantially in microbial composition at the phylum level (Figure 2A) and especially at the family level (Supplementary Figure S1). Of the 30,356 OTUs observed in >2 samples, 5,732 occurred in both soils, and 2,292 of these shared OTUs occurred in >2 samples from each soil. These shared OTUs were disproportionately abundant, comprising 36–66% (mean 50%) of sequences among soils, treatments, and timepoints (Figure 2B). Because soil type was the dominant factor influencing microbial community composition and obscured the effects of headspace treatments when all data were analyzed together, we first conducted separate ordinations for each soil to examine anoxic treatment impacts. Community composition varied predictably with anoxic duration and sampling date in both soils; these variables were strongly correlated with the first and second axes of the ordination, respectively (adjusted *R*^2^ = 0.45 and 0.47 for the Oxisol and Mollisol, respectively; *p* < 0.001 for anoxic duration and sampling date; Figures 3A,B). After 48 d, samples with shorter anoxic duration (0-day and 2-day treatments) remained clustered in ordination space near pre-treatment samples, whereas samples with increasingly longer anoxic duration diverged along the second ordination axis (Figures 3A,B). After 384 d, samples from all treatments shifted along the first axis but remained separated along the second axis according to anoxic duration (Figures 3A,B). A similar pattern was evident in a combined ordination of samples from both soil types which included only OTUs shared between the soils (Figure 3C). Samples separated along axes that were correlated with anoxic treatment duration and sampling date, albeit with lower R^2^ than in the separate ordinations (adjusted *R*^2^ = 0.13, *p* < 0.001 for sampling date and *p* = 0.006 for anoxic duration; Figure 3C).

The permutational multivariate ANOVA of the entire microbial community dataset measured after 384 d similarly indicated that soil type was the dominant predictor (*R*^2^ = 0.40, *p* < 0.0001), with a secondary role for anoxic treatment duration (*R*^2^ = 0.03, *p* = 0.005). After accounting for these variables, there were relatively weak relationships between community composition and cumulative CH_4_ production (*R*^2^ = 0.02, *p* = 0.02) and cumulative CO_2_ production (*R*^2^ = 0.01, *p* = 0.13). However, within individual soils, slightly stronger relationships emerged between community composition and C flux data. Cumulative CO_2_, cumulative CH_4_, and anoxic treatment duration had relatively similar marginal relationships to community composition in the Oxisol (*R*^2^ = 0.08, *p* = 0.01; *R*^2^ = 0.10, *p* = 0.003; *R*^2^ = 0.08, *p* = 0.01) and in the Mollisol (*R*^2^ = 0.07, *p* = 0.02; *R*^2^ = 0.09, *p* = 0.005; *R*^2^ = 0.14, *p* = 0.0002).

### Consistency of Responses to Anoxic Duration Among Taxonomic Groups

**Figure 4:**
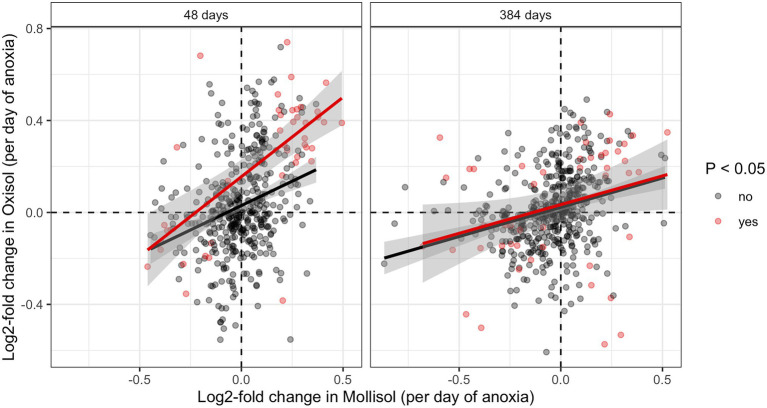
Pairwise comparisons of log2-fold change values calculated by DESeq for OTUs shared in both soils at 48 d or 384 d. Axis units are log2-fold change per day of anoxic period, so an OTU with a value of 0.083 d^−1^ would have an estimated log2-fold change of 1 in the 12-day anoxic treatment relative to the 0-day anoxic treatment (i.e., it doubled vs. the 0-day treatment). OTUs with statistically significant increases or decreases in both soils are shaded red (denoted “yes” in the legend); OTUs without a statistically significant change are shown in black (denoted “no” in the legend). Regressions including significant or non-significant OTUs are indicated by red and black lines, respectively (the former approach is more conservative; the latter is shown for completeness). The *R*^2^ and *p* values are as follows: 48 d, significant OTUs (*R*^2^ = 0.46, *p* < 0.0001); 48 d, non-significant OTUs (*R*^2^ = 0.09, *p* < 0.0001); 384 d, significant OTUs (*R*^2^ = 0.08, *p* = 0.05); 384 d, non-significant OTUs (*R*^2^ = 0.08 *p* < 0.0001).

The consistency of response of shared microbial groups to anoxic duration varied across taxonomic levels. At levels of phylum, class, and order, there was no significant overall shared response to anoxic duration in both soils. That is, increases or decreases in particular groups at these taxonomic levels as a function of anoxic duration were not shared between soils as indicated by pairwise comparisons of log2-fold change values (calculated by differential expression analysis) or by chi-squared tests for a similar direction of response (Supplementary Figure S2). However, we did observe a weakly significant anoxic response among shared families (*p* = 0.02, chi-squared test; Supplementary Figure S2D) and a strongly consistent anoxic response for shared OTUs (Figure 4). There were 88 combinations of OTUs and sampling dates where significant responses to anoxic duration occured in both soils (Figure 4), and 71 displayed a consistent positive or negative anoxic response in both soils—much greater than expected under the null hypothesis of independence (*p* < 0.0001, chi-squared test). Of these 71 OTU/sampling date combinations, 4 OTUs had significant responses to anoxic duration in both soils at both dates (48 and 384 d), such that 67 unique OTUs displayed consistent and significant responses to anoxic duration in both soils during our experiment (Supplementary Data Sheet S1). These OTUs were also disproportionately abundant, with six-fold greater (0.027%) median relative abundance than the other OTUs (0.0044%) in a given sample, considering all sequences measured at 48 d and 384 d (*p* < 0.0001). Considering all shared OTUs, log2-fold change values for anoxic duration response were also significantly correlated between soils (Figure 4; regression statistics are shown in the caption).

### Responses of Individual Taxonomic Groups to Anoxic Duration

**Figure 5:**
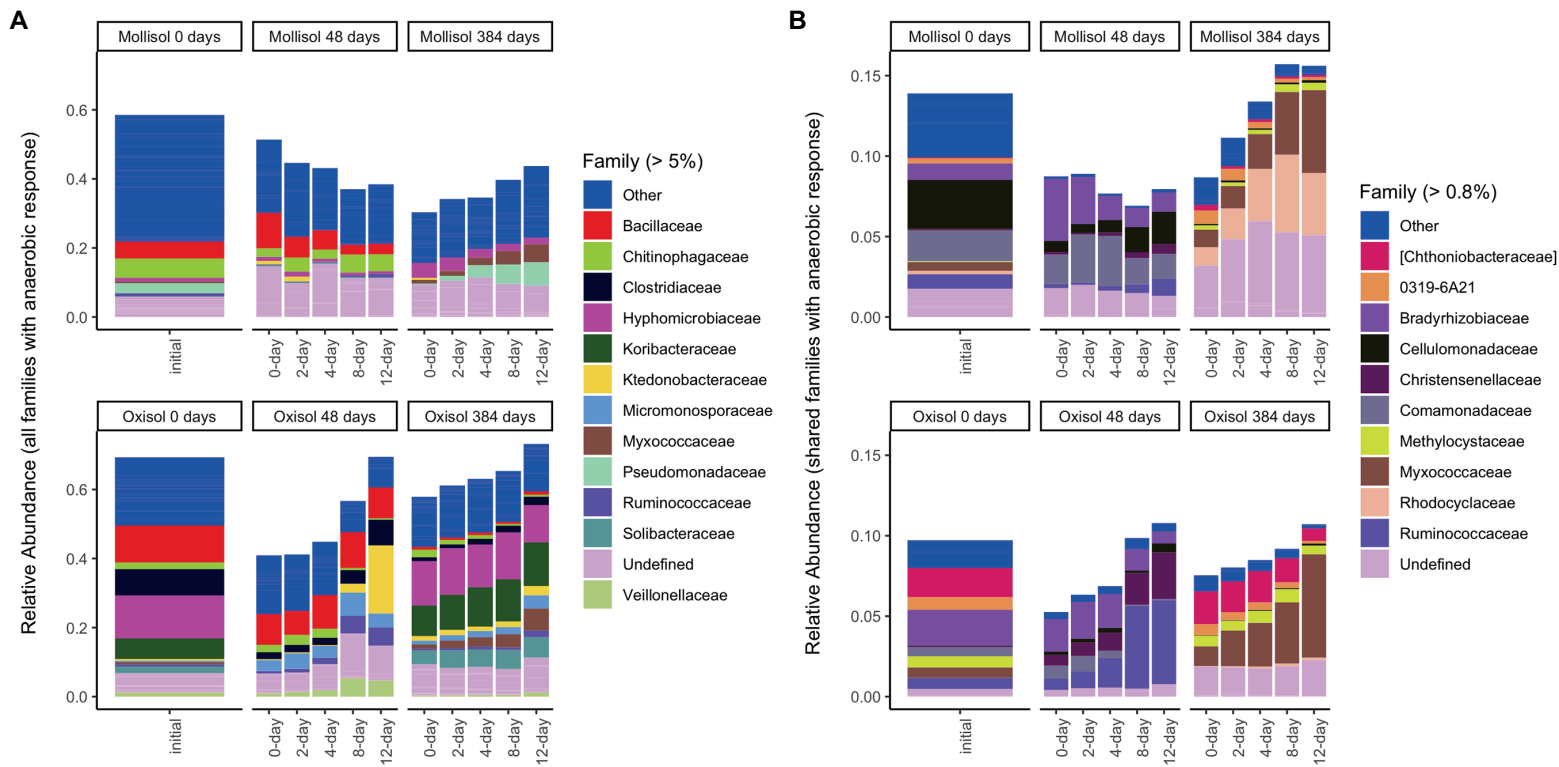
Mean sequence relative abundance by treatment, including all families that significantly responded to anoxic duration in a given soil/sampling date **(A)**, or only families with a significant response to anoxic duration that was consistent in both soils on a given date **(B)**. For clarity, only families comprising >5% **(A)** or 0.8% **(B)** of total sequences are labeled (other families are grouped as “Other”); un-named families are labeled as “Undefined.” Barplots of the initial (0-day) samples in **(B)** include sequences from families that responded significantly to anoxic duration after either 48 d or 384 d in the respective soil. Bars within a panel represent headspace treatment means (0, 2, 4, 8, or 12 d of anoxic conditions alternating with 4 d of oxic conditions).

Although many taxonomic groups did not show a consistent response to anoxic duration in both soils, many other groups did respond to anoxic duration in one soil at either 48 d or 384 d. Overall, sequences belonging to a group with a significant anoxic response accounted for 58% (phylum), 53% (class), 44% (order), 49% (family), and 34% (OTU) of sequences (Figure 5A; Supplementary Table S1). However, consistency of response of particular taxonomic groups to anoxic duration varied greatly between soils and over time. For example, the phylum *Bacteroidetes* increased with anoxic duration in the Mollisol but decreased in the Oxisol after 48 d, whereas *Firmicutes* showed no response in the Mollisol but strongly increased in the Oxisol (Figure 2). The responses of individual OTUs within these phyla generally corresponded to the phylum as a whole (Supplementary Figure S3). However, in contrast to the more abundant phyla, *Nitrospirae* and *Euryarcheaota* did respond strongly and consistently to anoxic duration in both soils after 384 d (decreasing and increasing, respectively; Supplementary Data Sheet S1). The *Euryarcheaota* that we detected in these soils were largely methanogens (class *Methanomicrobia*; Supplementary Figure S4).

Similar to the phylum level, responses to anoxic duration at the levels of class and order were also generally inconsistent between soils, with several exceptions (Supplementary Data Sheet S1). The class *OPB41* (*Actinobacteria*) increased with anoxic duration in both soils after 48 d and 384 d, *Anaerolineae* increased after 48 d, and *Solibacteres* and *Deltaproteobacteria* increased after 384 d. *Chthonomonadetes*, *Gemm-1* (*Gemmatimonadetes*), *PAUC37f* (*Acidobacteria*), and *OM190* (*Planctomycetes*) decreased with anoxic duration in both soils after 384 d. No orders responded consistently to anoxic duration after 48 d, but 15 responded after 384 d. In particular, orders belonging to different classes of *Proteobacteria* had consistent responses of differing direction: *Rhodocyclales* and *Myxococcales* increased with anoxic duration whereas *Rhodobacterales*, *Entotheonellales*, and *HTCC2188* decreased. Overall, classes with consistent responses to anoxic duration in both soils comprised 0.1–1.3% (mean 0.5%) of sequences after 48 d, increasing to 11.6–22.3% (mean 15.2%) after 384 d, while orders comprised 14.1–21.1% (mean 17.8%) after 384 d (Supplementary Table S1).

Families with a consistent response to anoxic duration had generally similar relative abundances at both 48 d and 384 d, comprising 5–16% (mean 9%) of sequences (Figure 4B; Supplementary Table S1; Supplementary Data Sheet S1). Two families responded consistently to anoxic duration in both soils at both timepoints: an unnamed family in *Solibacterales* decreased while *Cellulomonadaceae* increased. After 48 d, several families in *Proteobacteria* decreased with anoxic duration in both soils (*Bradyrhizobiaceae*, *Comamonadaceae*, *Haliangiaceae*, *Coxiellaceae*), whereas several families in *Clostridiales* increased (*Christensenellaceae*, *Peptococcaceae*, *Ruminococcaceae*). Of these families, only *Bradyrhizobiaceae* had similar relative abundance in both soils after 48 d; the other families tended to be much more abundant in one soil than the other (Figure 5B). After 384 d, several unnamed families in *Planctomycetes* decreased with anoxic duration in both soils, while families within *Acidobacteria*, *Actinobacteria*, and *Proteobacteria* showed both positive and negative responses. For example, within the *Alphaproteobacteria*, *Methylocystaceae* increased with anoxic duration while *Hyphomonadaceae* decreased. Of the relatively abundant families (> 0.8% of sequences) that responded consistently after 384 d, three had approximately similar relative abundances in both soils: *Myxococcaceae*, *Methylocystaceae*, and *0319-6A21*, a family in the *Nitrospirales* (Figure 5B).

OTUs with consistent responses to anoxic duration in both soils comprised a smaller proportion of total sequences than observed at the other taxonomic levels (1–8%, mean 4%), but responses between soils were more likely to be consistent for a given OTU (Figure 4). Four OTUs (two each in *Micromonosporaceae* and *Bradyrhizobiaceae*) decreased significantly with anoxic duration in both soils at both sampling dates, with log2-fold change values of −0.2 d^−1^ to −0.7 d^−1^ (Supplementary Data Sheet S1). These OTUs also had relatively similar mean abundances in the static oxic (0-day) treatment in both soils at both timepoints (0.01–0.04% of sequences). Of the many other OTUs that showed consistent responses to anoxic duration in both soils at either 48 d or 384 d, most belonged to the groups discussed above (Supplementary Figure S5; Supplementary Data Sheet S1). Several OTUs with a strong anoxic response in both soils were at least occasionally abundant, comprising >1% of sequences in a given sample. These included multiple OTUs in the genus *Dactylosporangium* (*Actinobacteria*, *Micromonosporaceae*) which responded positively, as well as in *Anaeromyxobacter* (*Deltaproteobacteria*, *Myxococcaceae*), *Candidatus* Koribacter (*Acidobacteria*, *Koribacteraceae*), and *Ruminococcaceae*. Three abundant *Sinobacteraceae* OTUs decreased with anoxic duration. OTUs from the Fe-reducing genus *Geobacter* showed a mixed response to anoxic duration (positive in the Mollisol and negative in the Oxisol), while OTUs from the putative Fe-reducing genus *Pelosinus* increased significantly with anoxic duration only in the Oxisol.

### Diversity and Evenness Responses to Anoxic Duration

**Figure 6:**
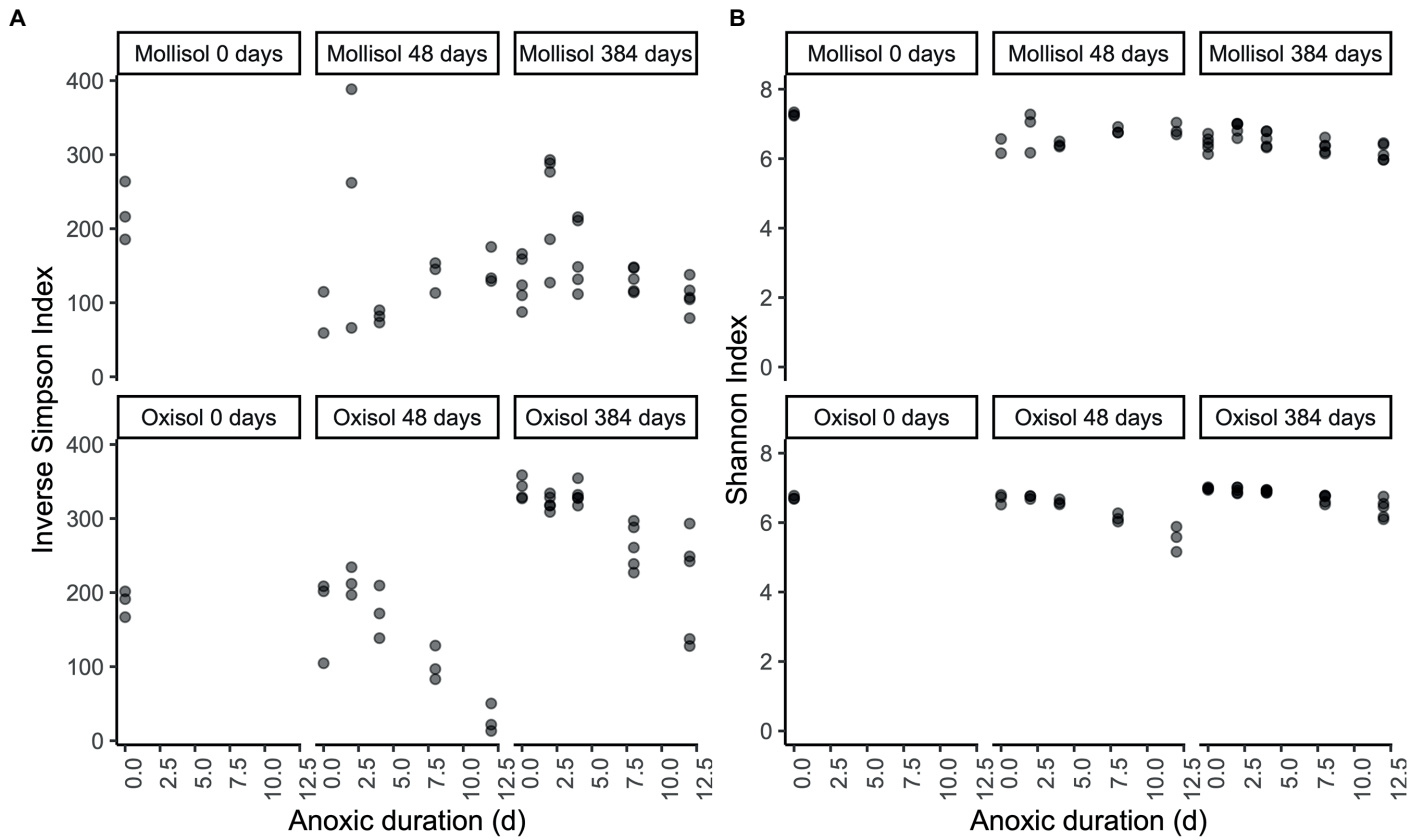
Inverse Simpson **(A)** and Shannon **(B)** diversity indices plotted by soil type and treatment. The x-axis indicates the duration of the anoxic period, plotted as a numeric variable (d). Panels represent sampling timepoints for each soil (0, 48, and 384 d), and x-axis values within a panel indicate the duration of the cyclic anoxic headspace treatment (0, 2, 4, 8, or 12 d of anoxic conditions alternating with 4 d of oxic conditions).

Inverse Simpson and Shannon Index values did not show consistent trends with anoxic duration throughout the experiment (Figure 6). In the Mollisol, inverse Simpson and Shannon Index values did not vary consistently with anoxic duration after 48 d. In the Oxisol, both indices decreased with anoxic duration after 48 d (*p* < 0.0001, linear regression), but the decreases were most pronounced in the 8- and 12-day anoxic treatments. After 384 d, inverse Simpson and Shannon Index values significantly (*p* < 0.05, linear regression) declined with anoxic treatment duration in both soils. However, in the Mollisol, indices actually tended to be higher in the 2- and 4-day anoxic treatments than the 0-d treatment (i.e., oxic control), and only declined consistently under the 8- and 12-day treatments. Similarly, in the Oxisol, these indices also tended to have similar values among the 0-, 2-, and 4-day treatments, and were only markedly lower in the 8- and 12-day treatments (Figure 6).

## Discussion

Supporting our first hypothesis, we found that both soils displayed large directional shifts in microbial community composition at multiple levels of taxonomic resolution when exposed to cyclic anoxic periods of increasing duration. Partially contradicting our second hypothesis, taxonomic groups occurring in both soils often responded differently to increased anoxic duration, particularly at coarse taxonomic resolution. However, in partial support of our second hypothesis, shared OTUs that did respond to anoxic duration had a strong tendency to respond in similar directions in both soils, and these 67 OTUs became disproportionately abundant during the experiment. Although the distinctly different initial communities in these two soils responded to O_2_ limitation in different ways, communities in both soils did vary systematically with increasing anoxic duration, and a core group of redox-sensitive taxa responded similarly even in the radically different environments (Table 1) of our rainforest Oxisol and cropland Mollisol.

### Systematic Community Responses to Anoxic Duration

A number of previous experiments have assessed impacts of fluctuating oxic/anoxic conditions on microbial community composition (Pett-Ridge and Firestone, 2005; DeAngelis et al., 2010; Frindte et al., 2016; Mejia et al., 2016; Randle-Boggis et al., 2018; Winkler et al., 2019; Pronk et al., 2020) but it was often difficult to discern whether increasing the relative durations of anoxic vs. oxic periods caused systematic changes, and whether these differences were sustained over time. Here, our treatments corresponded to cumulative anoxic durations of 0, 33, 50, 66, and 75% of the experiment. We found directional shifts in community composition at the level of OTUs (Figure 3) and in coarser taxonomic groups (Figures 1, 4) in both soils as anoxic period duration increased from 0 to 12 d. Temporal changes across all treatments were even larger than the effects of anoxic duration (Figure 3) and were likely influenced by gradual decreases in C availability and changes in C molecular composition over time (Huang et al., 2021), given the strong linkages between microbial composition and decomposition rate observed elsewhere (Fierer et al., 2007). Notably, however, the magnitude of community change with increasing anoxic duration was similar in both soils (Figure 3), and it persisted at both timepoints despite the large temporal change in community composition over the course of the 384-d experiment. This finding suggests that a generalizable imprint of periodic anoxia on microbial community composition, manifested by taxon relative abundance or perhaps taxon presence, might be detectable across diverse soils, even if it has a smaller overall effect than other controlling factors such as pH (Delgado-Baquerizo et al., 2018).

### Groups and Taxa That Consistently Responded to Anoxia

When high-level taxonomic groups responded to anoxic duration, responses generally differed between soils (Figure 2; Supplementary Figure S2) with a few notable exceptions. Consistent with their known physiologies, nitrifiers in the phylum *Nitrospirae* and methanogens in the class *Methanomicrobia* showed strong negative and positive relationships with anoxic duration, respectively, in both soils. Soil *Nitrospirae* also declined following a 6-week anaerobic disinfestation treatment (van Agtmaal et al., 2015); our results showed that even brief but periodic O_2_ deprivation can suppress these organisms in favor of anaerobes. The presence of *Methanomicrobia* in most soils and treatments (Supplementary Figure S4) implied that these organisms could either tolerate periodic O_2_ incursions or that anoxic microsites persisted throughout the experiment, consistent with observations of reduced Fe in all treatments (Huang et al., 2021) and gross methane production observed from other soils in oxic environments (von Fischer and Hedin, 2002). Putative aerobic methane-oxidizers (*Methylocystaceae*) also increased with anoxic treatment duration, implying that benefits of increased methane availability during the oxic periods outweighed stress during the anoxic period, possibly due to use of alternative metabolic pathways or formation of resting stages (Knief, 2019).

The general lack of consistent phylum-level responses to anoxic duration between soils resulted from contrasting responses of nested taxonomic groups, many of which did respond similarly in both soils. In particular, multiple *Actinobacteria* subgroups showed consistent positive or negative responses to anoxic duration. The uncultured class *OPB41* strongly increased with anoxic duration in both soils at both timepoints, and elsewhere this class co-occurred with methanogens in iron-rich aquatic environments (in’t Zandt et al., 2019) and in coal beds (Kirk et al., 2015). The *Actinobacteria* family *Micromonosporaceae* was highly sensitive to anoxic duration, and several OTUs showed consistent increasing or decreasing responses in both soils. The genus *Dactylosporangium* even contained multiple OTUs with differing anoxic responses (positive or negative) that were consistent in both soils. This putatively slow-growing genus was found to be abundant in hypoxic but not oxygenated lake water, possibly because of increased competitive ability under hypoxic conditions (de Menezes et al., 2012). The consistent sensitivity of these *Actinobacteria* classes, families, genera, and OTUs to anoxic duration across our two contrasting soils highlights the possible ecological importance of this group and its potential utility as an indicator of soil redox history.

Similar to the *Actinobacteria*, the *Acidobacteria* also showed strong but occasionally differing responses to anoxic duration at multiple taxonomic levels. The class *Solibacteres* and order *Solibacterales* increased with anoxic duration in both soils after 384 d; these groups occurred in other variable redox environments including Arctic peat (Lipson et al., 2013) and wastewater treatment bioreactors (Zhang et al., 2017) and increased after a pasture soil was flooded (Randle-Boggis et al., 2018). Two OTUs in the genus *Candidatus* Solibacter decreased with anoxic duration at 48 d. Genomic analyses suggest that these organisms are slow-growing aerobic heterotrophs (Ward et al., 2009), but they became increasingly abundant in paddy soils following long-term organic matter amendments (Yu et al., 2019), implying that they required time to proliferate under the conditions of our experiment. *Solibacterales* and *Koribacteraceae* also increased in agricultural soils under continuous corn cultivation, which was interpreted by the authors as a response to nutrient limitation (Soman et al., 2017). The strong response of these groups to anoxic duration in our experiment suggest that differences in anoxic microsites between cropping systems might also have contributed. *Candidatus* Koribacter may oxidize metals at redox interfaces (Ward et al., 2009; Allward et al., 2018), and this group increased with anoxic duration after 384 d in both of our soils, indicating their possible role in oxidizing reduced iron (Fe) or manganese during the oxic phases.

Both soils reduced and oxidized Fe at rates of tens to hundreds of μg Fe per g soil per anoxic/oxic cycle, as indicated by comparisons of Fe^II^ concentrations between oxic and anoxic treatment phases during the first 48 d of the experiment (Huang et al., 2021). The 16S data implicated several taxa that might have contributed to Fe reduction. *Deltaproteobacteria* showed contrasting responses among subgroups but *Myxococcaeae* and 10 OTUs from *Anaeromyxobacter* increased strongly with anoxic duration in both soils. This genus contains taxa capable of dissimilatory Fe reduction as well as aerobic respiration (Treude et al., 2003) and it increased elsewhere following Fe addition to rice paddy soils (Wang et al., 2020). *Myxococcales* also increased under redox fluctuation treatments in other experiments (DeAngelis et al., 2010; Randle-Boggis et al., 2018; Winkler et al., 2019). In a separate soil sample collected at the same site where our Puerto Rican Oxisol was obtained, meta-transcriptomic analyses indicated that *Geobacter*, *Pelosinus*, and *Anaeromyxobacter* were likely the most important Fe-reducing organisms (Wilmoth et al., 2018). However, in our Oxisol samples, *Geobacter* OTUs were rare, similar to the results of DeAngelis et al. (2010), who also used samples from this same site. *Pelosinus* OTUs were present in both soils but only significantly responded to anoxic duration in the Oxisol. The relatively high abundance of *Anaeromyxobacter* OTUs and their consistent increase with anoxic duration suggest that they may have contributed to measured Fe reduction in both soils.

Microbes that increased with anoxic duration in our experiment often corresponded with groups identified previously in wetland soils, anaerobic digestors, and in anaerobic disinfestation experiments (where soils were amended with C substrates and covered with impermeable material to kill pathogens). Several *Ruminococcaceae* OTUs increased with anoxic duration, consistent with their dominance following reductive disinfestation (Huang et al., 2019) and their increase in rice-paddy soil following straw amendment (Wegner and Liesack, 2016). The *Chloroflexi* class *Anaerolineae* increased with anoxic duration; this group was abundant in rice-paddy soil under current-producing conditions (Cabezas et al., 2015) or following iron mineral addition (Huang et al., 2020a), and was a core member of anaerobic digestor communities (Rivière et al., 2009). Taxa that responded negatively to anoxic duration included multiple classes and orders in the *Planctomycetes* which declined following anaerobic disinfestation (van Agtmaal et al., 2015) and in anoxic horizons of tundra soils (Lipson et al., 2015). Several *Bradyrhizobiaceae* OTUs consistently decreased with anoxic duration, and this broader group (*Rhizobiales*) also decreased in flooded pasture soils (Randle-Boggis et al., 2018). The functional attributes of these particular *Bradyrhizobiaceae* are unclear, but because of the abundance and ubiquity of this group here and elsewhere (Delgado-Baquerizo et al., 2018) they deserve attention as indicators of O_2_ availability.

## Conclusion

### Some Core Taxa May Respond Consistently to Periodic Oxygen Limitation

Oxygen availability has been proposed as a core driver of soil microbial community composition (Fierer, 2017) but we are unaware of experiments that explicitly tested the impacts of temporal gradients in O_2_ availability in diverse environments. Using two soils with greatly differing biological and geochemical characteristics, we identified a core guild of O_2_-sensitive microbes across multiple taxonomic levels. Responses were most consistent between soils at the OTU level. Shifts in the abundance and presence of the particular taxa identified may therefore hold promise in recording the recent history of O_2_ availability. Many O_2_-sensitive OTUs identified here were also common and prevalent members in global soil communities (Delgado-Baquerizo et al., 2018; Supplementary Data Sheet S1). It remains to be seen, however, whether these taxa also might consistently respond to O_2_ variation in other soils, and experiments comparing a broader range of soils are needed to expand on the proof-of-concept experiment we report in this paper. Although O_2_ tolerance/sensitivity may be a strongly conserved trait for some taxonomic groups, other groups showed disparate responses to anoxic period duration at relatively fine taxonomic resolution (Supplementary Data Sheet S1), indicating a potential for adaptation or niche partitioning. Furthermore, the O_2_-sensitive OTUs identified here which were not commonly or consistently observed in previous syntheses of soil microbes may also prove useful in delineating soils that experience periodic O_2_ deficiency from consistently oxic environments. Notably, shared responses of OTUs to O_2_ deficiency occurred despite large differences between our study soils in pH and other environmental attributes (Table 1) and their greatly contrasting initial community compositions (Figure 2). Along with variables such as pH, temperature, C availability, and moisture (Fierer and Jackson, 2006; Fierer et al., 2007; Delgado-Baquerizo et al., 2018; Frindte et al., 2019), our results support the hypothesis (Fierer, 2017) that time-integrated O_2_ availability might serve as a core driver of microbial composition across diverse terrestrial soils from humid environments. Given the tremendous spatiotemporal heterogeneity of O_2_ in terrestrial soils and the challenges of directly measuring this key biogeochemical variable at scales relevant to microbial physiology (Sexstone et al., 1985; Silver et al., 1999; Hall et al., 2013; Keiluweit et al., 2016), microbial community datasets deserve further attention as proxies for redox history.

## Data Availability Statement

The datasets presented in this study can be found in online repositories. The names of the repository/repositories and accession number(s) can be found at: https://www.ncbi.nlm.nih.gov/, PRJNA693044; https://doi.org/10.6073/pasta/1ee938a080a0000dca8c138add8f9930, edi.1125.1.

## Author Contributions

WH and SH designed the experiment. WH conducted the experiment. SN and ER performed bioinformatic analyses. SH analyzed the OTU data and wrote the paper with contributions from all authors. All authors contributed to the article and approved the submitted version.

## Funding

This research was supported in part by NSF grants DEB-1457805 and EAR-1331841. Funds for 16S rRNA gene amplicon sequencing were provided by a University of Wisconsin-Madison Microbiome Initiative award to EER. The open access publication fees for this article were paid by the Iowa State University Library.

## Conflict of Interest

The authors declare that the research was conducted in the absence of any commercial or financial relationships that could be construed as a potential conflict of interest.

## Publisher’s Note

All claims expressed in this article are solely those of the authors and do not necessarily represent those of their affiliated organizations, or those of the publisher, the editors and the reviewers. Any product that may be evaluated in this article, or claim that may be made by its manufacturer, is not guaranteed or endorsed by the publisher.
