# Supplementary material for: Shared Microbial Taxa Respond Predictably to Cyclic Time-Varying Oxygen Limitation in Two Disparate Soils
Source: Front Microbiol. 2022 Jun 2;13:866828. doi: 10.3389/fmicb.2022.866828 (PMC9203030; doi:10.3389/fmicb.2022.866828)
Supplement: Supplementary file 2 [file Data_Sheet_1.pdf]

Supplementary Material for: Shared microbial taxa respond predictably to cyclic time-varying oxygen limitation in two disparate soils

Steven J. Hall[1*], Wenjuan Huang[1], Stephanie A. Napieralski[2], Eric Roden[2]

[1]Department of Ecology, Evolution and Organismal Biology, Iowa State University, Ames, IA, USA

[2]Department of Geoscience, University of Wisconsin-Madison, Madison, WI, USA

*** Correspondence:**
Steven J. Hall
stevenjh@iastate.edu

The Supplementary Information contains Supplementary Methods, 2 Tables, and 5 Figures on 9 pages. The column descriptions for Supplementary Data S1 are also provided here.

Supplementary Methods: Construction and Sequencing of v3-v4 16s Metagenomic libraries

Purified genomic DNA was submitted to the University of Wisconsin-Madison Biotechnology Center. DNA concentration was verified fluorometrically using either the Qubit® dsDNA HS Assay Kit or Quant-iT™ PicoGreen® dsDNA Assay Kit (ThermoFisher Scientific, Waltham, MA, USA). Samples were prepared according to Illumina's 16s Metagenomic Sequencing Library Preparation Protocol, Part # 15044223 Rev. B (Illumina Inc., San Diego, California, USA) with modifications as follows. The 16S rRNA gene V3/V4 variable region was amplified with fusion primers (forward primer 341f: 5'-ACACTCTTTCCCTACACGACGCTCTTCCGATCT(N)$_{0/6}$<u>CCTACGGGNGGCWGCAG</u>-3', reverse primer 805r: 5'-GTGACTGGAGTTCAGACGTGTGCTCTTCCGATCT(N)$_{0/6}$<u>GACTACHVGGGTATCTAATCC</u>-3'). Region specific primers (underlined sequences above) were previously described in Klindworth et al., (2013), and were modified to add 0 or 6 random nucleotides ((N)$_{0/6}$) and Illumina adapter overhang nucleotide sequences 5' of the gene-specific sequences. Following initial amplification, reactions were cleaned using a 0.7x volume of AxyPrep Mag PCR clean-up beads (Axygen Biosciences, Union City, CA). In a subsequent PCR, Illumina dual indexes and Sequencing adapters were added using the following primers (forward primer: 5'-ATGATACGGCGACCACCGAGATCTACAC[5555555555]ACACTCTTTCCCTACACGACGCTCTTCCGATCT-3', Reverse Primer: 5'-CAAGCAGAAGACGGCATACGAGAT[7777777777]GTGACTGGAGTTCAGACGTGTGCTCTTCCGATCT -3', where bracketed sequences are 10bp custom Unique Dual Indexes). Following PCR, reactions were cleaned using a 0.7x volume of AxyPrep Mag PCR clean-up beads (Axygen Biosciences). Quality and quantity of the finished libraries were assessed using

an Agilent 4200 TapeStation DNA 1000 kit (Agilent Technologies, Santa Clara, CA) and Qubit® dsDNA HS Assay Kit (ThermoFisher Scientific), respectively. Libraries were pooled in an equimolar fashion and appropriately diluted prior to sequencing. Paired end, 300 bp sequencing was performed using the Illumina MiSeq Sequencer and a MiSeq 600 bp (v3) sequencing cartridge. Images were analyzed using the standard Illumina Pipeline, version 1.8.2.

References:

Klindworth, A., Pruesse, E., Schweer, T., Peplies, J., Quast, C., Horn, M., et al. (2013). Evaluation of general 16S ribosomal RNA gene PCR primers for classical and next-generation sequencing-based diversity studies. *Nucleic Acids Research* 41, e1. doi: 10.1093/nar/gks808.

Table S1: Mean proportions of sequences belonging to a group with a significant response to anoxic duration, listed by soil, treatment (length of anoxic period, in days), and sampling date (0, 48, or 384 d). "Consistent" groups are those that shared a directional response in both soils at a given date. For the "Initial" treatment (i.e., the pre-treatment samples), sequences which showed a significant response at either of the subsequent sampling dates (48 or 384 d) are included.

Soil	Treatment	Sampling date (d)	All phyla	Consistent phyla	All classes	Consistent classes	All orders	Consistent orders	All families	Consistent families	All OTUs	Consistent OTUs
Mollisol	Initial	0	0.504	0.005	0.667	0.055	0.544	0.089	0.585	0.139	0.312	0.016
Mollisol	0	48	0.109	NA	0.329	0.005	0.337	NA	0.514	0.087	0.272	0.033
Mollisol	2	48	0.1	NA	0.349	0.007	0.281	NA	0.446	0.089	0.215	0.017
Mollisol	4	48	0.122	NA	0.267	0.005	0.265	NA	0.431	0.077	0.178	0.01
Mollisol	8	48	0.19	NA	0.26	0.011	0.177	NA	0.37	0.069	0.182	0.011
Mollisol	12	48	0.22	NA	0.248	0.013	0.17	NA	0.384	0.079	0.205	0.018
Mollisol	0	384	0.476	0.009	0.466	0.116	0.317	0.152	0.303	0.087	0.433	0.043
Mollisol	2	384	0.435	0.008	0.513	0.129	0.376	0.205	0.341	0.111	0.426	0.038
Mollisol	4	384	0.438	0.004	0.522	0.144	0.379	0.209	0.346	0.134	0.406	0.03
Mollisol	8	384	0.511	0.003	0.538	0.14	0.39	0.206	0.397	0.157	0.461	0.029
Mollisol	12	384	0.537	0.002	0.533	0.138	0.395	0.197	0.437	0.156	0.504	0.028
Oxisol	Initial	0	0.948	0.008	0.823	0.079	0.765	0.073	0.693	0.097	0.376	0.041
Oxisol	0	48	0.745	NA	0.429	0.001	0.339	NA	0.409	0.053	0.248	0.027
Oxisol	2	48	0.736	NA	0.424	0.001	0.324	NA	0.411	0.063	0.259	0.034
Oxisol	4	48	0.739	NA	0.451	0.002	0.382	NA	0.448	0.069	0.249	0.034
Oxisol	8	48	0.811	NA	0.601	0.001	0.494	NA	0.566	0.099	0.391	0.063
Oxisol	12	48	0.873	NA	0.718	0.002	0.649	NA	0.694	0.108	0.535	0.055
Oxisol	0	384	0.885	0.007	0.772	0.148	0.656	0.141	0.579	0.075	0.325	0.049
Oxisol	2	384	0.889	0.005	0.78	0.155	0.679	0.147	0.611	0.08	0.307	0.048
Oxisol	4	384	0.885	0.005	0.782	0.159	0.697	0.149	0.631	0.085	0.31	0.049
Oxisol	8	384	0.904	0.004	0.794	0.171	0.709	0.161	0.653	0.092	0.341	0.058
Oxisol	12	384	0.937	0.002	0.852	0.223	0.793	0.211	0.732	0.107	0.467	0.08

Table S2: Identifying information for sequence files deposited in the NCBI Sequence Read Archive under accession PRJNA693044.

SRA sample	Soil	Treatment	Day of experiment
1	Oxisol	initial	0
2	Oxisol	initial	0
3	Oxisol	initial	0
4	Oxisol	12-day	48
5	Oxisol	12-day	48
6	Oxisol	12-day	48
7	Oxisol	8-day	48
8	Oxisol	8-day	48
9	Oxisol	8-day	48
10	Oxisol	4-day	48
11	Oxisol	4-day	48
12	Oxisol	4-day	48
13	Oxisol	0-day	48
14	Oxisol	0-day	48
15	Oxisol	0-day	48
16	Oxisol	2-day	48
17	Oxisol	2-day	48
18	Oxisol	2-day	48
19	Mollisol	initial	0
20	Mollisol	initial	0
21	Mollisol	initial	0
22	Mollisol	12-day	48
23	Mollisol	12-day	48
24	Mollisol	12-day	48
25	Mollisol	8-day	48
26	Mollisol	8-day	48
27	Mollisol	8-day	48
28	Mollisol	4-day	48
29	Mollisol	4-day	48
30	Mollisol	4-day	48
31*	Mollisol	0-day	48
32	Mollisol	0-day	48
33	Mollisol	0-day	48
34	Mollisol	2-day	48
35	Mollisol	2-day	48
36	Mollisol	2-day	48
37	Mollisol	0-day	384
38	Mollisol	0-day	384
39	Mollisol	0-day	384
40	Mollisol	0-day	384
41	Mollisol	0-day	384
42	Mollisol	2-day	384
43	Mollisol	2-day	384
44	Mollisol	2-day	384
45	Mollisol	2-day	384
46	Mollisol	2-day	384
47	Mollisol	4-day	384
48	Mollisol	4-day	384
49	Mollisol	4-day	384
50	Mollisol	4-day	384

51	Mollisol	4-day	384
52	Mollisol	8-day	384
53	Mollisol	8-day	384
54	Mollisol	8-day	384
55	Mollisol	8-day	384
56	Mollisol	8-day	384
57	Mollisol	12-day	384
58	Mollisol	12-day	384
59	Mollisol	12-day	384
60	Mollisol	12-day	384
61	Mollisol	12-day	384
62	Oxisol	0-day	384
63^	Oxisol	0-day	384
64	Oxisol	0-day	384
65	Oxisol	0-day	384
66	Oxisol	0-day	384
67	Oxisol	2-day	384
68	Oxisol	2-day	384
69	Oxisol	2-day	384
70	Oxisol	2-day	384
71	Oxisol	2-day	384
72	Oxisol	4-day	384
73	Oxisol	4-day	384
74	Oxisol	4-day	384
75	Oxisol	4-day	384
76	Oxisol	4-day	384
77	Oxisol	8-day	384
78	Oxisol	8-day	384
79	Oxisol	8-day	384
80	Oxisol	8-day	384
81	Oxisol	8-day	384
82	Oxisol	12-day	384
83	Oxisol	12-day	384
84	Oxisol	12-day	384
85	Oxisol	12-day	384
86	Oxisol	12-day	384

*this sample had poor DNA quality and was not included

^this sample was not used in the analysis because of low read count

Supplementary Data S1: This file contains the microbial groups which had a consistent response to anoxic treatment duration in both soils after 48 d or 384 d, according to DESeq analysis. The columns denoted “basemean” for the Oxisol and Mollisol show the means of the normalized count values, divided by size factors (Love et al., 2014), for these respective soils. The columns denoted “log2foldChange” are the log2-fold increase or decrease of a taxon, expressed per day of anoxic treatment duration (i.e., this value would be multiplied by 12 for the 12-day anoxic treatment). The columns denoted “padj” indicate the adjusted P values of the response to anoxic treatment duration for the particular taxon and soil. The column “Present in synthesis of Delgado-Baquerizo et al. 2018” indicates whether a particular taxon occurred frequently across the diverse terrestrial soils measured by those authors.

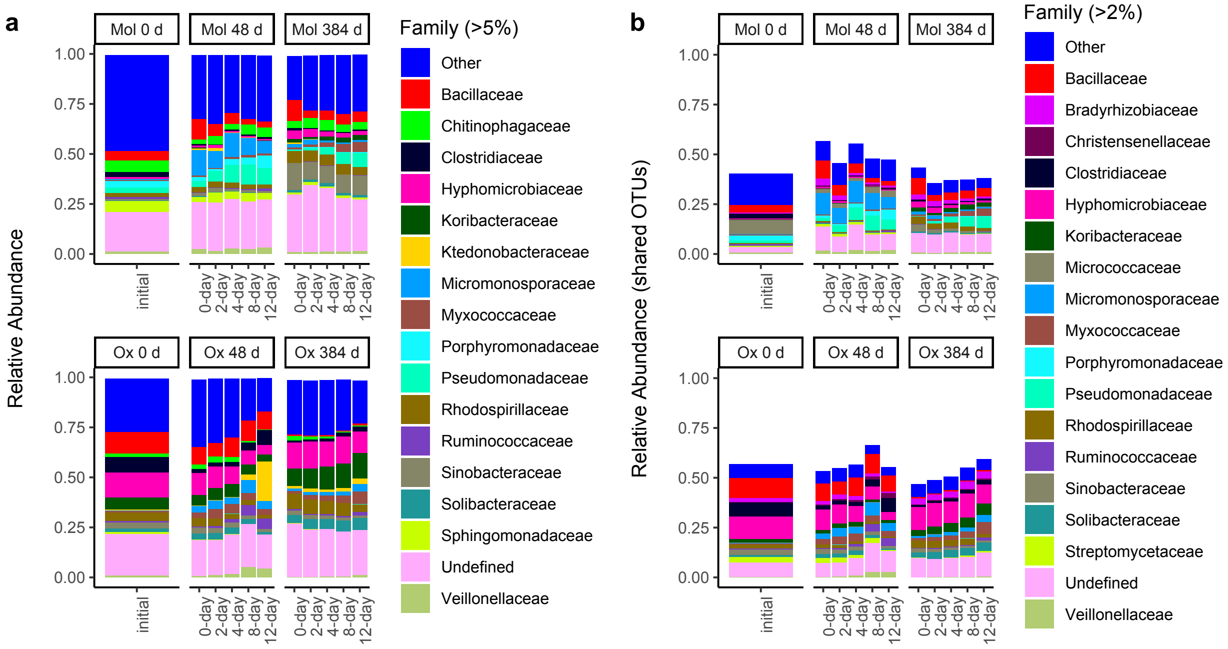

Figure S1: Stacked barplots of OTU relative abundance by family, including all OTUs from families which comprised > 5% of sequences in a given sample (a), or only OTUs that occurred in both soils, for families that comprised > 2% of sequences in a given sample (b). The panel abbreviations "Mol" and "Ox" denote the Mollisol and Oxisol, respectively. Panels represent sampling timepoints for each soil (0, 48, and 384 d), and bars within a panel are headspace treatment means (0, 2, 4, 8, or 12 d of anoxic conditions alternating with 4 d of oxic conditions).

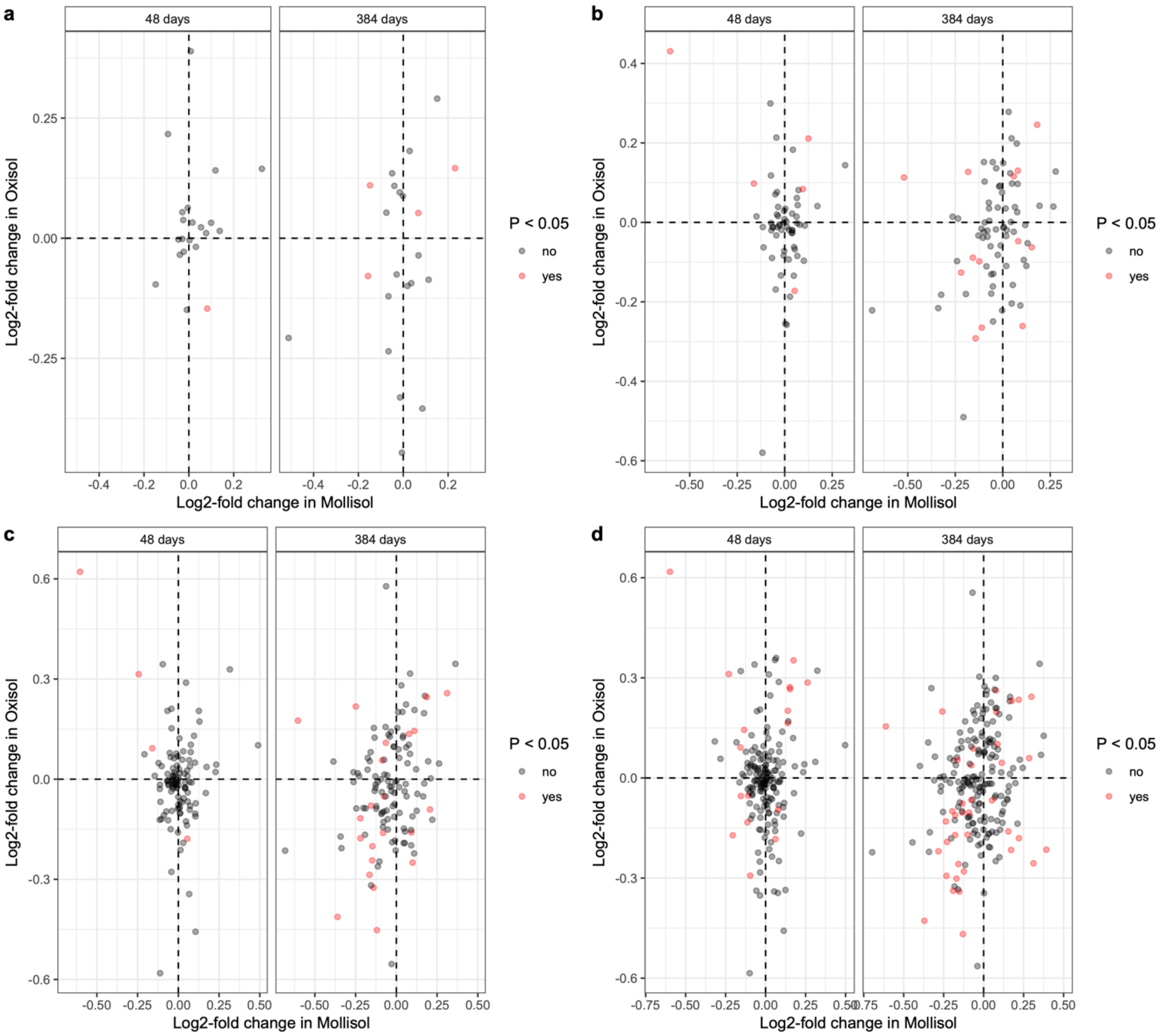

Figure S2: Pairwise plots of log2-fold change values for anoxic duration comparing microbial groups that occurred in both the Mollisol and Oxisol, at different taxonomic levels: (a) phylum, (b) class, (c) order, and (d) family. Axis units are log2-fold change per day of anoxic period duration, so an OTU with a value of 0.083 d^{-1} would have an estimated log2-fold change of 1 in the 12-d anoxic treatment relative to the control (i.e., it approximately doubled). Red circles indicate OTUs where log2-fold change values were statistically significant in both soils.

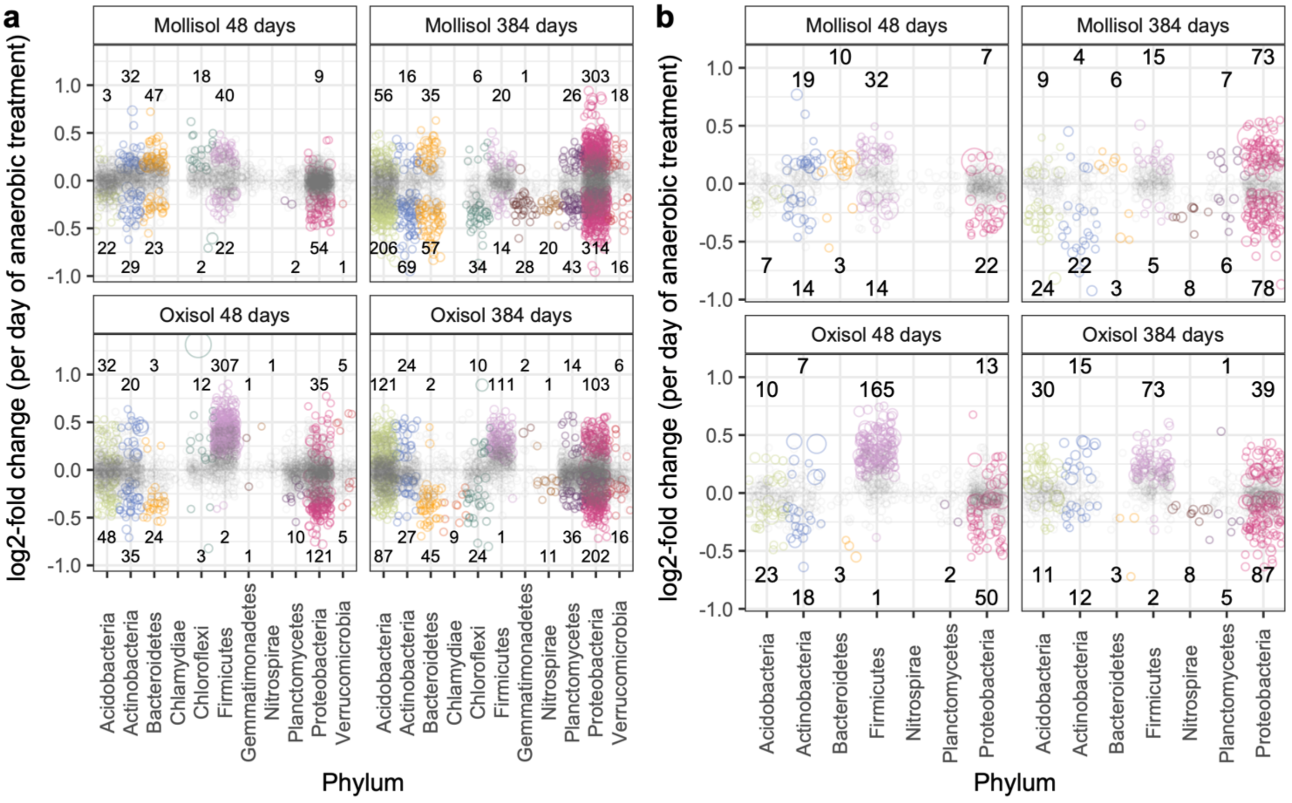

Figure S3: Changes in OTU relative abundance (log2-fold changes) as a function of anoxic treatment duration, plotted by phylum (x axis). Panels represent sampling timepoints for each soil (48 and 384 d). The OTUs with a statistically significant response to anoxic treatment duration are indicated by colored circles, and non-significant OTUs are shown in grey. The number of OTUs in each phylum which significantly increased or decreased with anoxic treatment duration is respectively indicated above or below the point cloud. All phyla with > 5 OTUs that were significantly impact by anoxic duration at a given soil/sampling date are shown, except for phylum WS3 (which only varied with anoxic duration in the Oxisol at 384 d).

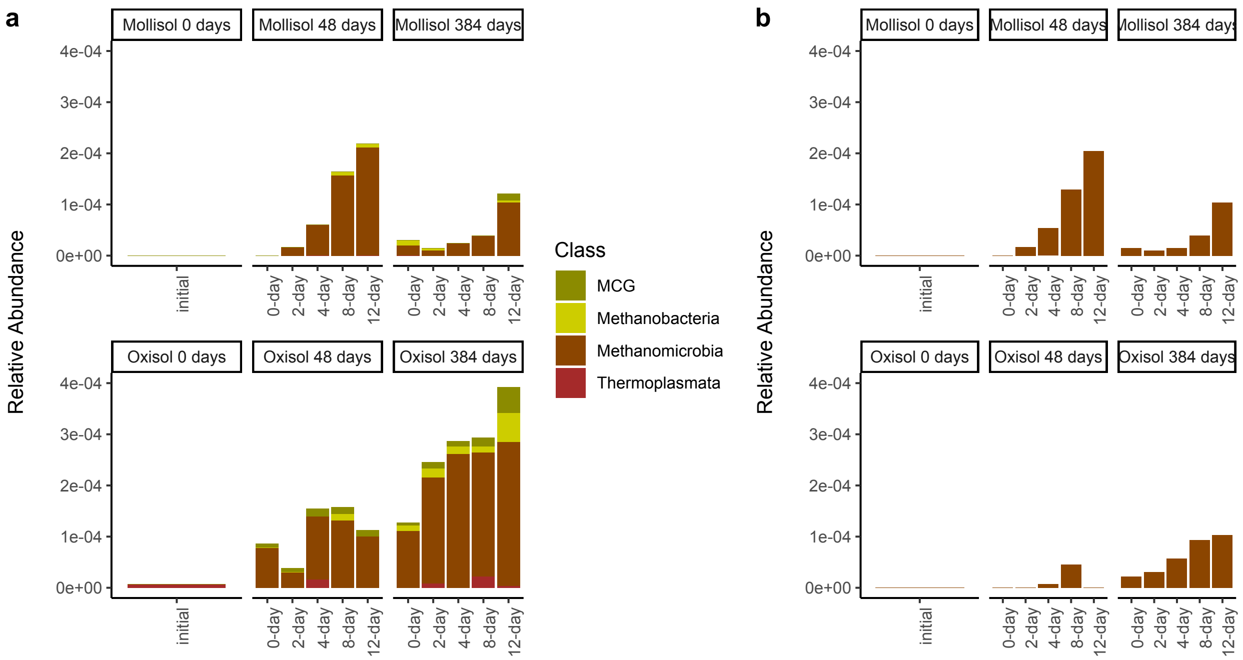

Figure S4: Stacked barplots of archaea OTU relative abundance by class, including all archaea classes in a given sample (a), or only OTUs that occurred in both soils (b). Panels represent sampling timepoints for each soil (0, 48, and 384 d), and bars within a panel represent means of headspace treatments (0, 2, 4, 8, or 12 d of anoxic conditions alternating with 4 d of oxic conditions).

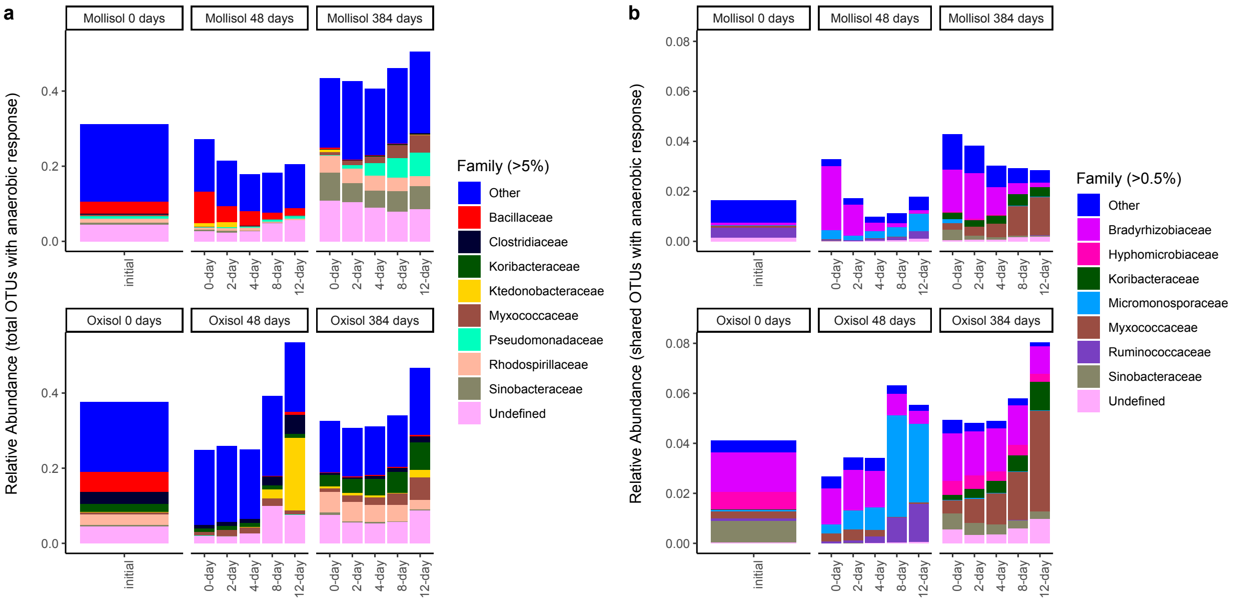

Figure S5: Stacked barplots of OTU relative abundance by treatment and shaded by family, including all OTUs that significantly responded to anoxic duration in a given soil/sampling date (a), or only OTUs that had a significant response to anoxic duration that was consistent in both soils on a given sampling date (b). For clarity, only OTUs from families comprising > 5% (a) or 0.5% (b) of total sequences are labeled (other families are grouped as "Other"); OTUs belonging to un-named families are labeled as "Undefined". Barplots of the initial (0-day) samples include sequences from OTUs that responded significantly to anoxic duration after 48 d or 384 d in the respective soil. Bars within a panel represent means of respective headspace treatments (0, 2, 4, 8, or 12 d of anoxic conditions alternating with 4 d of oxic conditions).
